# A perspective on methodologies and system boundaries to develop abatement cost for on-farm anaerobic digestion

**DOI:** 10.1080/21655979.2023.2245991

**Published:** 2023-09-15

**Authors:** Jorge Diaz Huerta, Richard O’Shea, Jerry Murphy, David M. Wall

**Affiliations:** a MaREI Centre, Environmental Research Institute, University College Cork, Cork, Ireland; b Civil, Structural and Environmental Engineering, School of Engineering and Architecture, University College Cork, Cork, Ireland

**Keywords:** Anaerobic digestion, techno-economic assessment, abatement cost, GHG mitigation, life-cycle analysis

## Abstract

Marginal Abatement Cost Curves compare and assess greenhouse gas mitigation options available to various sectors of the economy. In the Irish agricultural sector, large anaerobic digestion facilities are currently considered a high-cost abatement solution. In prior studies of anaerobic digestion abatement costs, two options were assessed: the generation of heat and electricity from biogas (115 €/tCO_2eq_) and the production of renewable heat from biomethane (280 €/tCO_2eq_). Both scenarios encompass single cost values that may not capture the potentially variable nature of such systems. In contrast, prior techno-economic analyses and lifecycle analyses can provide a comparison of the abatement costs of anaerobic digestion systems at a range of scales. This work compares two case studies (based on prior literature) for small and medium-scale on farm anaerobic digestion systems. The small-scale system is set in Ireland with cattle slurry collected in open tanks during the winter, while the medium-scale system is set in the USA with cattle slurry collected periodically indoors all year-round. It was found that the abatement cost can vary between −117 to +79 € per t CO2_eq_. The key variables that affected the abatement cost were additional revenue streams such as biofertilizer sales, displaced energy savings, and additional incentives and emissions savings within the system boundary. Including only some of these options in the analysis resulted in higher abatement costs being reported. Based on the variation between system topologies and therefore system boundaries, assigning a single mitigation cost to anaerobic digestion systems may not be representative.

## Highlights

- The veracity of an abatement cost analysis depends on a clear methodological process.
- The abatement cost varies based on the processes considered within the system boundary.
- On-farm digestion abatement costs assessed ranged from -117 to +79 €/tCO_2eq_.
- On-farm emissions savings ranged from 609 to 10,358 tCO_2eq_/yr.
- Abatement costs reduce when considering the income and emissions savings from co-benefits.

## 1. Introduction

With the increasing threat of global warming due to rising greenhouse gas (GHG) emissions, climate mitigation has become a leading priority for the European Union (EU). Through the European Parliament’s environmental policies, a goal has been set to reduce GHG emissions in all sectors of the economy and to achieve carbon neutrality by 2050 [1]. In 2019, the EU emitted a total of 4,057 Mt of carbon dioxide equivalent (CO_2eq_), of which the agriculture sector was responsible for 11% [2]. In agriculture, there are limited mitigation options that can reduce GHG emissions and improve manure and livestock management while reducing waste production [3]. Mitigation options can be limited to reducing the demand of consumers for meat and dairy products or converting arable land to permanent pasture or wood land to sequester carbon in the soil [4]. Technologies such as anaerobic digestion (AD) can provide a manure management option that mitigates GHG emissions by converting manures and slurries into biogas that can replace fossil fuels, as well as reducing fugitive methane emissions from manure storage [5]. On-farm AD may be a suitable mitigation option in Ireland owing to the wide availability of agricultural biomass. With over 90% of agricultural land covered by grass, excess grass, which is not used as livestock fodder, could provide a potential feedstock to produce biogas in Ireland [6]. Furthermore, the livestock sector produces a significant share of agricultural waste in the form of manure (or slurry) produced by ca. 7 million cattle [7] in a country with a population of ca. 5 million people. Previous studies have found that grass silage should be co-digested with slurry to ensure sufficient nutrients are available to enable the effective production of biogas [8].

On-farm AD can facilitate the progression to a sustainable agricultural sector by processing agricultural waste (such as slurry) on-site, reducing the overall emissions from manure management, and providing an indigenous renewable energy source in the form of biogas. AD simultaneously provides energy and fuel generation whilst reducing water, soil, and air pollution [9]. There are drawbacks associated with anaerobic digestion such as fugitive methane emissions (ranging from 2% to 10%) from different sources within the process (including biogas production, upgrading to biomethane, and digestate storage) [10]. These can be minimized through careful design and prudent operation practices (such as the implementation of covered digestate storage) [11]. The biofertiliser (digestate) produced as a by-product of AD can displace raw manure (slurry) use on farms and reduce the associated risk of water contamination and GHG emissions. Digestate use also mitigates odors associated with manure storage and decomposition whilst removing pathogens [12]. Pyrolysis of digestate may also be implemented to produce biochar that can be used as an additive to improve the digestion process and boost the overall biogas production, or it can be spread on the land as a soil amendment facilitating carbon sequestration and net zero carbon emissions within the process [13].

To successfully implement anaerobic digestion projects an accurate prediction of the economic viability which takes into consideration the ever-changing process characteristics is required. Techno-economic assessments (TEAs) can determine the potential technical and financial performances and the overall feasibility of projects. Such models consist primarily of mass and energy balances of the proposed system with the financial data related to the cost and revenues generated during the lifetime of the project [14]. Scenario-type analyses within a TEA allow for a high-level assessment of the variation and feasibility of AD plants using sensitivity analysis. This can be coupled with a Life-Cycle Assessment (LCA) to compare and analyze the impact of different technologies and products on the environment based on the carbon emissions generated or avoided during the entire life cycle of a process. The impact of the process can be analyzed in terms of the inputs and outputs of the materials and the energy used in the process/product life cycle [15]. A critical aspect for the development of an LCA is the definition of the boundary for the analysis [16]. Based on the inputs and outputs selected, different system boundaries can be drawn, each one leading to different results and a multitude of scenarios. The results of both analyses (TEA and LCA) can be combined to measure the mitigation potential of a technology and compared to other technologies to provide a better understanding of the options available.

A marginal abatement cost curve (MACC) can be a useful tool to evaluate the cost and abatement potential of various mitigation technologies. This curve orders the options based on the cost of GHG abatement of a measure, and by the quantity of GHG emissions mitigated [17]. Abatement measures are ranked based on the cost-effectiveness the technology provides in a specific sector, and the results of these rankings have been used by many governments to establish low carbon policies [18]. In Ireland’s *Climate Action Plan 2019* [19], a MACC was used to assess the impact of technology adoption across all sectors of the economy to reach the decarbonization target of 30% GHG emissions reduction in the non ETS sector by 2030. Furthermore, a specific MACC for the agriculture sector was developed to compare different energy mitigation measures (Table 1) [20]. Some of the measures considered for GHG mitigation included: the reduction of energy consumption on dairy farms (Farm Energy); the increase in biomass use from sources such as wood thinnings and sawmill (Forestry); the use of willow and miscanthus to produce heat (Biomass-Heat); the use of willow chips and peat to generate electricity (Biomass-Electricity); production of biodiesel from oilseed rape to substitute fossils fuels imports (biodiesel); and production of bioethanol from sugar beet to substitute fossils fuel imports (bioethanol).

**Table 1.** MACC results for the agriculture sector of Ireland for 2021–2030. Adapted from [20].

Scenario	Scenario description	Marginal abatement cost (€/t CO_2eq_)	Abatement potential (kt CO_2eq_)
Farm Energy	Increased farm energy efficiency through energy consumption reduction on-site.	−359	29
Forestry (Wood/Wood Residue)	Use of fuel-wood, sawmill residues and waste wood for energy generation.	−30.7	759
Biomass (Heat)	Increased use of short rotation coppice and miscanthus biomass for heat production.	−20	179
Biomass (Electricity)	Combustion of willow and miscanthus in biomass boilers for energy generation.	−10	196
Biodiesel	Biodiesel production from oilseed rape.	90	174
Biogas AD	Biogas production from co-digestion of slurry and grass silage for energy generation.	115	224
Bioethanol	Bioethanol production from sugar beet.	200	51
Biomethane	Biomethane production from biogas upgrading for grid injection.	280	150

As part of the analysis, two anaerobic digestion options were considered as potential mitigation options within the agriculture and forestry sectors as shown in Table 1. The first option was related to the production of biogas via the digestion of slurry and grass silage to generate heat and electricity (115 €/tCO_2eq_). The second option was to produce biomethane from biogas, followed by the use of this biomethane to produce renewable heat (280 €/tCO_2eq_). Both options were seen as high-cost abatement solutions for the Irish agricultural sector based on a price point of 50 €/tCO_2eq_ [20]. MACC analyses are dependent on the methodology used and the inputs considered for the calculations. As such, the MACC may not capture the entire cost-effectiveness of the technology depending on the methodology used to calculate the mitigation potential [17]. Furthermore, MACC analyses have largely been completed for large-scale operations that encompass whole energy sectors, where little granularity of the technologies or options available are provided [21,22]. As a result, mitigation options such as anaerobic digestion have been represented by a single value related to a single scenario that may not encompass or describe the impact of different variables that can change between different systems (based on scale, type, or energy output). Anaerobic digestion systems vary in scale, design, technology, feedstock, and function, and these variabilities cannot be covered by a single scenario within a MACC.

This study aims to provide a perspective on previous methodologies used for assessing the economic and environmental feasibility of on-farm anaerobic digestion systems. To the best of the authors’ knowledge no prior literature has outlined the importance of the assumptions made in the calculation of the abatement potential and associated cost for on-farm anaerobic digestion systems. Thus, this work will advance beyond the state of the art by encompassing a more holistic methodology for the calculation of the abatement cost of a technology such as anaerobic digestion. To represent all the potential costs and benefits that a technology may provide, based on its abatement cost and potential, clarity on the variables and assumptions selected for the construction of the abatement curve is required. For this work, methodologies used for calculating the cost and abatement potential of on-farm anaerobic digestion systems will be reviewed. The abatement cost of on-farm anaerobic digestion will be assessed for two case studies at different scales. Results will be compared against the values provided for anaerobic digestion in the Climate Action Plan shown in Table 1. Furthermore, the main variables and assumptions required to calculate the mitigation potential of on-farm anaerobic digestion and their impact on results will be explored.

## 2. Methodologies

### 2.1. Areas of focus within the system boundary of an on-farm AD system

The AD process at a farm-scale can be divided into three main areas of focus for an economic analysis: 1) feedstock production/acquisition and transport including the operations and costs associated with the collection and transportation of feedstock to the AD plant; 2) pre-treatment and AD processes including the capital and operational costs of processing the feedstock and conversion to biogas and digestate, and 3) biogas post-processingutilization and coproduct handling including but not limited to the cost of upgrading the biogas and the handling of the digestate produced from the AD process [23]. The processes associated with each area of focus may vary depending on the design of an individual system. Table 2 highlights previous literature from a select set of works of TEA and LCA analyses which includes a description of the aforementioned areas of focus. These studies were used as they all included at least one process relating to the three areas of focus of the AD process outlined above. A full list of the articles reviewed can be found in detail in Appendix A. Providing a system boundary facilitates the development of a process model and calculation of the mitigation potential, cost, and revenue associated with each area.

**Table 2.** Areas of focus in previous literature studies including TEA and LCA analyses of on-farm AD systems.

Areas of Focus	Associated Processes	TEA						TEA + LCA			LCA		
Feedstock production/Acquisition and Transport	Feedstock Acquisition	X	X	X	X	X	X	X	X	X	X		
	Feedstock Handling	X	X	X			X	X	X	X	X		X
	Manure and/or Feedstock Storage							X	X	X	X	X	X
	Transportation by Land or Pipe	X						X	X	X	X		X
	Farm Labor		X					X		X			
Pre-treatment and Anaerobic Digestion	Construction of Anaerobic Digester	X	X	X	X	X	X	X	X	X	X	X	X
	Purchase of CHP unit	X	X	X	X	X	X	X	X	X	X	X	X
	Acquisition of Heat Exchange and Water Pumps	X	X	X	X		X		X				
	Maintenance and Repair of the System	X	X	X	X		X	X	X	X			
	Insurance			X	X			X	X	X			
	Plant Labor		X	X	X		X	X	X	X			
Biogas Post Processing/Utilization and coproduct handling	Connection to Gas Grid or Electricity Grid					X							X
	Purchase of Gas Purification Unit					X							X
	Operation of Upgrading System					X							X
	Sale of Electricity and/or Heat Generated	X	X	X	X	X	X	X	X	X	X	X	X
	Sale of Biomethane Produced					X							
	Land Spreading of Digestate	X		X	X	X	X	X			X	X	
	Treatment of Digestate						X			X		X	
	Sale of Digestate as Biofertilizer		X				X	X	X		X	X	
Source		[24]	[25]	[26]	[27]	[28]	[29]	[30]	[31]	[32]	[33]	[34]	[35]

X marks studies that contain the variables or process detailed in the leftmost column of the table.

**Figure 1.**
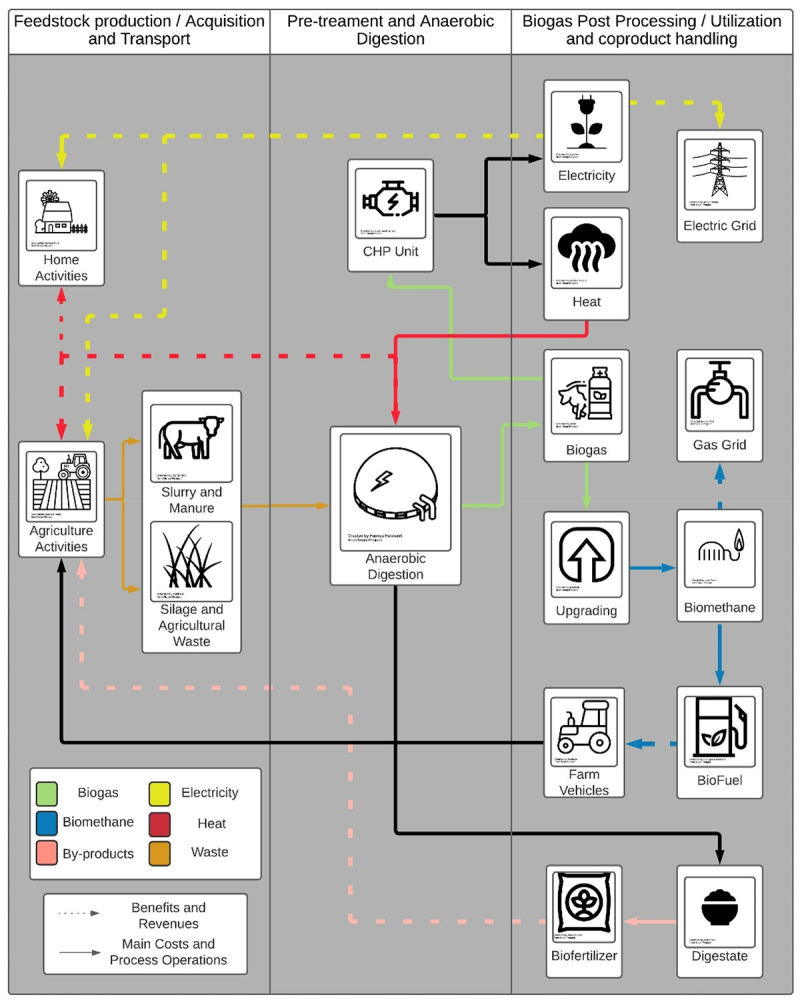
Example of an on-farm AD system, including the costs and revenues taken with an economic diversification approach. Elements of the graphics sourced from the Noun Project [37].

For TEA and LCA, the selection of the processes within the system boundary determines the scope of the analysis and can affect the estimates of operating cost, capital cost, revenues, and the total carbon abatement of the overall process. Furthermore, process models need to be created around this methodology to include all the relevant variables based on the farm and AD system for which the analysis is being undertaken. A representative farm model, which captures the entire farm context of the region of analysis, is preferred to account for all inputs and outputs considered for the TEA and LCA. This can be quite extensive based on the data and resources required to determine the multitude of interacting factors that might impact upon the viability of an AD system in a real-world context. However, once the estimations are set, the constructed model can be easily adapted to a variety of farm types and scales [36]. For that reason, a methodology in which the processes considered for the analysis are clear, such as the one shown in Figure 1, for the region of analysis facilitates the use of a single modeling tool that can replicate different scenarios and compare the mitigation potential taking into consideration variations in the calculation of the abatement cost.

### 2.2. Capital and operational costs of on-farm AD

In a TEA, the costs can be divided into the cost of capital expenditure (CapEx) and operational expenditure (OpEx). For on-farm AD, the main capital expenditure can be designated to the investment required for the plant design which in turn is defined by the type of feedstock used, the digester size desired, and the available technology [38]. Once the type of plant has been selected, the total construction budget is divided into costs such as the engineering and permits required for its construction, construction insurance, interconnection costs, developer’s fee, equipment acquisition/installation costs, and land acquisition [39]. Furthermore, depending on the business model there may be additional capital expenses including for a CHP unit, digestate treatment systems, and an upgrading unit to convert the biogas to biomethane [40]. CapEx costs can also vary depending on the region where the technology is applied as some of the equipment may need to be imported from another country, incurring additional costs in the form of taxes.

For OpEx, the associated costs are the operation and maintenance of the AD plant. These costs can include the daily operational labor, acquisition of feedstock, chemical and consumables required for the system, insurance, taxes, and maintenance of the mechanical systems [39]. Depending on the type of feedstock, the cost associated with the operation can increase if there is a need for additional processes such as waste screening, removal of contaminants, and the need for loading and unloading of feedstock based on the size and type of digester [38]. Furthermore, if the system is not correctly maintained, over time there may be a need to replace some of the mechanical and electrical elements of the system. It is also important to take into consideration an operation and maintenance (O&M) reserve fund to cover unexpected costs in the project such as the need to replace any equipment due to malfunctions [39]. Finally, the cost of the parasitic electrical and thermal energy demand based on the selected AD technology must also be considered. For a process model to have an accurate estimation of the CapEx and OpEx, it is important to consider all associated processes of the technology that will be considered as part of the economic evaluation of the AD system as opposed to part of the farm function. For most of the TEAs and LCAs reviewed in the literature, these costs are considered within the areas of focus of the AD process as shown in Table 3.

**Table 3.** Capital and operational costs for on-farm AD systems reported in previous literature considering TEA and LCA analysis.

Costs		TEA							TEA + LCA			
Capital	Plant Construction	X	X	X	X				X		X	
	Construction Insurance or Contingency								X			
	Grid Connection Cost					X						
	Equipment Acquisition/Installation Cost	X	X	X	X	X	X	X	X	X	X	X
	Land Acquisition and/or Civil Works		X	X	X		X	X	X		X	X
	Upgrading Unit	X				X						
Operational	Operational Labor	X	X				X		X	X	X	X
	Acquisition of Feedstock		X	X			X	X			X	X
	Chemicals and Consumables for the System					X	X		X		X	
	Insurance for the System			X	X					X	X	
	Internal Energy Consumption		X	X	X	X	X		X	X	X	X
	Maintenance	X	X	X	X		X	X	X	X	X	X
	Handling of Feedstock	X					X	X			X	X
	Digestate Treatment		X				X				X	
Source		[24]	[25]	[26]	[27]	[28]	[29]	[41]	[42]	[30]	[31]	[32]

X marks that the study contains the variables or process detailed in the left side of the table.

### 2.3. Economic diversification and revenues from on-farm AD

The main revenues obtained from on-farm AD are dependent on the post-processing and utilization of biogas. A CHP unit fueled by biogas can generate renewable electricity and heat that can be sold to generate revenues. In the case of electricity, these revenues come from Feed-in-Tariffs (FiT) set by regulators within countries or regions that are contract-based with a maximum duration of 15–20 years. Different electricity prices are based on the sources of biomass or the capacity of the plant as shown in Table 4. In the case of heat, revenues can be generated through renewable heat incentives (RHI), such as those used in the UK and Ireland that are tier-based depending on the AD plant size, and similar to FIT tariffs are available for 15–20 years. A caveat in reading Table 4 is the ever-changing systems for reimbursement in different countries over time. The values presented illustrate typical ways of remunerating renewable energy at a moment in time for selected countries. However, it can be noted that the incentive typically increases when the system scale is small, thus encouraging the adoption of small-scale on-farm AD systems. Such granularity of incentives is considered crucial by numerous governments to enable a profitable and competitive market for biogas plants [47], whilst encouraging returns to the sector at a local level which cannot avail of large (industrial) economies of scale. However, if additional products and benefits are provided by an on-farm AD system over time and are integrated into the farm supply chain, the process can increase the potential for financial sustainability without the need for subsidies.

**Table 4.** Financial benefits and support payments for AD in selected countries. Adapted from Pablo-Romero et al. [44], RES Legal [43] and RHI from the UK and Ireland [45,46].

Country	Contract Duration*	Determining Factors of the Prices	Revenue from Biogas	Source
Germany	20 years	Plant size and origin of fuel (biogas from bio-waste, manure, landfill, sewage gas)	**Biomass from bio-waste**: €ct 13.05–14.88 per kWh **Biogas from manure**: €ct 23.13 per kWh	[43]
United Kingdom	20 years	Installed capacity of the plant.	**Plant Capacity**: **≤250 kWe**: €ct 8.83 per kWh **>250 kWe and ≤500 kWe**: €ct 8.15 per kWh **>500 kWe**: €ct 8.4 per kWh **Anaerobic Digestion Heating Systems**: **Capacity <**200 MWth: 4.83 GBP (in pence) **Capacity** ≥200 kWth - <600 kWth: 3.79 GBP (in pence) **Capacity** ≥600 kWth: 1.21 GBP (in pence)	[44,45].
France	15 years	The capacity of the plant and the energy performance	**Plant Capacity**: **≤150 kWe**: €ct 9.745 per kWh **≥2 MWe**: €ct 8.121 per kWh A bonus of €ct 4 is granted to the plant if at least 60% or more of the biomass contains livestock manure.	[44]
Ireland	15 years	Technology dependent (landfill gas, anaerobic digestion) and size of the CHP or non-CHP plant	**Anaerobic digestion CHP plant capacity (No longer open for applications)**: **CHP≤500 kWe**: €ct 15.7 per kWh **CHP>500 kWe**: €ct 13.66 per kWh **Non-CHP≤500 kWe**: €ct 11.55 per kWh **Non-CHP>500 kWe**: €ct 10.5 per kWh **Anaerobic Digestion Heating Systems**: **Tier 1** (0–300 MWh/yr): 2.95 €ct/kWh **Tier 2** (300–1,000 MWh/yr): 2.95 €ct/kWh **Tier 3** (1,000–2,400 MWh/yr): 0.50 €ct/kWh	[44,46].

*Period guaranteed by each government to offer fixed prices per kWh generated.

Based on the processes considered in the business model selected for the on-farm AD system, the sources of revenues may vary between different TEAs, as shown in Table 5. The main source of income for most on-farm AD systems is the sale of electricity generated by the CHP unit. However, depending on the region of analysis, an economic diversification approach may be applied. This approach considers the generation of multiple products, which can provide benefits in terms of cost reduction to the process and additional income streams [48]. This concept for on-farm AD is extended to other potential outputs within the boundary of the on-farm AD process that are non-energy related.

**Table 5.** The main revenues generated from on-farm AD systems reported in the previous literature considering TEA and LCA analysis.

Revenues	TEA						TEA + LCA		
Sale of electricity to the grid	X	X	X	X	X	X	X	X	X
Sale of biogas and/or biomethane	X				X	X			
Sale of heat in the district heating network							X		X
Sales of by-products from digestate or co-products from the system	X							X	X
Sales of biofertilizer (digestate)	X	X				X			
Offset of current Heat and/or Electricity costs avoided from current farm demand*.		X	X	X			X		X
Source	[24]	[25]	[26]	[27]	[28]	[29]	[30]	[31]	[32]

X marks that the study contains the variables or process detailed in the left side of the table.

*This stream is considered a revenue as it reduces the cost of farm processes not related to the AD process.

The inclusion of the digestate (biofertilizer) and volatile fatty acids could diversify the product portfolio of the AD industry to increase its viability and feasibility. For example, carbohydrates present in the liquid effluent may be used to produce carboxylic acids by using membrane filtration which can be sold as platform chemicals [49]. Digestate can be converted to bio-oil and bio-char with the integration of pyrolysis. Through the use of novel upgrading technologies, biogas can be converted into methanol, bio-CNG, and/or liquid drop-in fuels [50]. Biomethane can also be used as a vehicle fuel with the use of compressed natural gas (CNG) service stations [51]. With the increase in support schemes and technical advancements, there has been an increase in natural gas vehicles (NVGs) in the EU; ca. 1.3 million vehicles were reported in operation in 2015. Up to 3,345 CNG filling stations, of which 697 were biomethane filling stations, have been reported in selected EU countries (Germany, Italy, Austria, Finland, Sweden, Iceland, and Norway) [12]. Biomethane can also be a substitute for natural gas if it is injected into the gas grid for use by traditional end-users (power plants, industries, and households). However, for this to be possible, the biomethane needs to comply with the gas purity requirements of the relevant gas grid.

The sources of income or revenues may vary between different TEAs, this is dependent on the business model selected and the processes considered for the analysis, as shown in Table 5. Furthermore, if the uncertainty of external variables which can vary between regions is added, affecting both the cost and revenues that can change with time, the results can vary between each study. To account for this variability, some TEAs include additional sensitivity analysis to assess which variables are more sensitive to external variables within the study scope.

### 2.4. Sensitivity analysis and the variability in costs and revenues

Sensitivity analyses are used to evaluate the uncertainty that independent variables may pose to dependent variables in a mathematical model based on a series of assumptions. This allows for the identification of the input factors that contribute to the uncertainty of the model for later optimization [52]. For investment projects, it is essential to add a sensitivity analysis as an evaluation procedure to assess the feasibility of the project. This includes input values (such as the income, costs, value of investments), and how external factors may affect the total investment project evaluation [53]. To assess the impact of external factors within the study scope on the profitability of on-farm AD, it is necessary to assess changes in the inputs and outputs of the systems that directly affect CapEx and OpEx during the lifetime of the project. Such external factors include government energy policies in the region and the technological development of AD at the time of the analysis, which directly affects the economic performance of the plant [54].

In a TEA, sensitivity analysis can be conducted to assess the impact of the OpEx and the revenues on the key parameters such as the payback period, profitability index, and the net present value [55]. However, depending on the type of analysis, the investment capital related to the CapEx may be included in the sensitivity analysis if different technologies or systems are evaluated in the process [29]. Some examples of the OpEx considered include operating capacity, power efficiency, electricity price, gas price, feedstock price (manure, silage, or other agricultural feedstock), farm size, pre-treatment costs, and discount rate as shown in Table 6. For the revenues, these variables may include applied subsidies, electricity, and heat buyback price, solid digestate price, liquid effluent price, biomethane price, liquid, and solid fertilizer price (Table 6). Once the variables are identified, they are altered by a set variation value to identify the variables that have the greatest impact on the output(s) and the results can be visualized using a tornado plot. This allows for the identification of the external agents that are most relevant for uncertainty analysis using a Monte Carlo simulation which is conducted by repeatedly running the model with different values of the sensitive parameters sampled from a given probability distribution [52].

**Table 6.** Variables evaluated for sensitivity analysis in recent TEA literature studies.

Variables analyzed in Sensitivity Analysis	TEA				TEA + LCA
Operating Capacity			X		X
Feedstock Price	X	X		X	X
Farm Size		X	X		
Capital Cost	X		X	X	X
Pre-treatment Costs		X			
Discount Rate		X	X		
Applied Subsidies				X	
Electricity and Heat Buyback Price		X	X	X	
Digestate and Effluent Price					X
Fertilizer Price				X	
Source	[24]	[26]	[27]	[29]	[31]

X marks that the study contains the variables or process detailed in the left side of the table.

### 2.5. Implementation of TEA and LCA for on-farm AD

An indication of the financial feasibility of on-farm AD can be estimated from the results of recent TEA scenarios undertaken in different regions across the globe. These results tie together the processing capacity of the plant selected for the analysis based on the CapEx, OpEx, and the break-even prices of the products considered within the AD process in the region of analysis. The most prominent key indicators included in the results of recent TEA analyses include the IRR, NPV, and the payback period as shown in Table 7. The IRR plays a crucial role in assessing the viability and financial feasibility of the project before investing. The NPV provides the present value of all future cash flow the project will generate during the lifetime of the project, which in most of the TEAs reviewed for on-farm AD is in the range of 15–30 years. Each TEA selects a particular discount rate based on the assumptions of the inflation in the region of analysis, the weighted average cost of capital of the investor, and investor attitudes toward risk; typically, this rate is in the range of 5% to 10%, although it is commonly recommended to select a discount rate of 10% for renewable energy investments if there are no previous data for the region [56]. Furthermore, most of the TEAs reviewed provide a simple payback period, in comparison to a discounted payback period, which provides a more realistic representation of the payback period by taking into consideration the value of money over time.

**Table 7.** Key indicators reported by recent TEA analyses in the literature for on-farm AD.

TEA Key Indicators	Khan et al. (2014) [24]	Wresta et al. (2015) [25]	Imeni et al. (2019) [26]	Imeni et al. (2019) [27]	Perta et al. (2019) [28]	Villarroel-Schneider et al. (2020) [29]	O’Connor et al. (2019) [30]	Aui et al. (2019) [31]
IRR	X	X	X	X	X		X	X
NPV	X	X	X	X	X	X	X	X
LCOE	X					X		
Simple Payback Period	X	X	X	X	X	X	X	X
Discounted Payback Period						X	X	

X marks that the study contains the variables or process detailed in the left side of the table.

In terms of LCAs, most studies follow the International Organization for Standardizations (ISO) 14040 and 14,044 standards [31] although, for Europe, the International Reference Life Cycle Data System (ILCD) handbook guide provides a detailed description on how to carry out the analysis following the definitions established by the European guidelines [32]. This includes the emissions analysis that needs to be set based on the REDII methodology to assess GHG emissions from the production and use of biomass-derived fuels; for the co-digestion of crop biomass this would include everything from the cultivation of the crop right through to biogas upgrading and/or injection for use as a transport fuel as shown in Eq. 1. If the fuel is used for heat and/or electricity production, an energy conversion factor needs to be included to account for the efficiency of the heat or electricity generation process when comparing the energy produced to the respective fossil fuel comparator.

1 $$E=\overset{n}{\underset{1}{\sum }}S_{n}\times \left(e_{ec,n}+e_{td,feedstock,n}+e_{l,n}-e_{sca,n}\right)+e_{p}+e_{td,product}+e_{u}-e_{ccs}-e_{ccr}$$

Where E amounts to the total emissions produced in the biogas as primary energy (before energy conversion to electricity, heat, or transport fuel); $S_{n}$ is the energy share provided by feedstock n for the production of the biofuel, $e_{ec,n}$ is the emissions associated with the extraction and cultivation of feedstock n; $e_{td,feedstock,n}$is the emissions from the transport of feedstock n to the digester; $e_{l}$ is the emissions from carbon stock changes caused by land-use change; $e_{p}$ is the emissions from processing; $e_{td}$ is the emissions from transport and distribution of the products; and $e_{u}$ is the emissions from fuel in use, emitted during combustion, which shall be assumed as 0 for biofuels and bioliquids. The emissions savings are accounted for by $e_{sca}$ from improved agricultural management of feedstock, $e_{ccs}$ and $e_{ccr}$ from CO_2_ capture from geological storage and fuel replacement [57].

In the case of co-product allocation in which one or more useful products are produced in the production system (such as heat, transport fuel, biofertilizer, etc.) it is necessary to select an allocation method so that the emissions can be divided between the co-products [35]. To achieve this, some authors recommend expanding the system boundary of the LCA to include the life-cycle of co- or by-products to correctly represent the environmental performance of the biofuel system [58]. Furthermore, the LCA boundary determines the mitigation potential of the on-farm AD system based on the GHG abatement mitigation measures considered in the boundary as shown in Table 8.

**Table 8.** The main GHG mitigation measures taken into consideration for literature LCAs for on-farm AD.

Life-cycle	O’Connor et al. (2019) [30]	Aui et al. (2019) [31]	Croxatto Vega et al. (2020) [32]	Nayal et al. (2016) [33]	Pardo et al. (2017) [34]	Adams et al. (2019) [35]
Manure Management	X		X	X	X	X
Displacement of Farm Energy Demand	X	X	X	X		
Displacement of Energy Exported	X			X	X	X
Displacement of Mineral and Organic Fertilizers		X		X	X	

X marks that the study contains the variables or process detailed in the left side of the table.

### 2.6. Abatement cost curve methodology

With the values obtained from both the TEA and LCA, it is possible to calculate the abatement cost that can assess the impact of technology adoption compared to other technologies in the agricultural sector based on the abatement cost and the magnitude of abatement possible. The abatement cost can be calculated using the IPCC methodology [15] as shown in Eq. 2.

2 $$Abatement\;\;cost=\frac{-NPV}{DLA}$$

Where the NPV is obtained from the result of the TEA and the discounted lifetime abatement (DLA) is calculated from the sum of the discounted annual abatement (DA) as shown in Eq. 3.

3 $$DLA=\overset{n}{\underset{t=0}{\sum }}DA_{t}=\overset{n}{\underset{t=0}{\sum }}\frac{Net\;GHG\;Abatement}{{\left(1+r\right)}^{t}}$$

Where the *Net GHG Abatement* (tCO_2eq_./yr) is obtained from the result of the LCA, *r* is the discount rate, *t* is the year of analysis, and *n* is the number of years based on the lifetime of the project.

An abatement cost analysis evaluates the potential for GHG mitigation based on two main variables: the cost of implementing the measure and the effectiveness in reducing the emissions in the sector. Based on Sathaye and Meyers [59], abatement cost curves (ACCs) follow three typical approaches.

The partial approach evaluates a measure against a reference technology in which the incremental changes in emissions and costs are calculated and ranked based on the cost-effectiveness of the measure. The results are ranked independently without taking into consideration the possible interdependence among the options making this approach the shortest and simplest; however, this can sometimes cause misinterpretation of the results.

The retrospective approach is similar to the partial approach in the initial steps, but multiple calculations are required to evaluate each option or measure based on the interdependence of the variables present and the interaction these have with all of the previously implemented options. Compared to the partial approach, it is slower as it requires more calculations and does not consider the interaction between more expensive options on previously selected options.

The integrated system approach requires a well-defined baseline and the use of an optimization model that reacts automatically to marginal changes in the demand and supply side every time the model is run. This approach is the most complete option as it considers all possible interdependencies within the system boundary of the analysis.

MACC analyses for the agriculture sector have been developed for several countries where AD is proposed as a mitigation measure to reduce GHG emissions. For the majority of MACCs that include AD as a mitigation technology, the preferred methodology is the partial approach [20,60,61], despite being more prone to misinterpretations due to the lack of data availability used for its construction. A recent MACC [62] used a retrospective approach to evaluate the interdependence of the variables within the analysis. The abatement cost and potential can be quite dependent on the region of analysis, the size of the farm, the type of feedstock, and the possibility to commercialize the co-benefits. Thus, the selection of all the possible variables affecting the system under analysis is essential in terms of considering all measures that contribute to the mitigation potential of the on-farm AD system.

### 2.7. Considerations for the analysis of abatement cost for on-farm AD

For the majority of TEAs and LCAs reviewed, the processes and variables selected were dependent on the region of analysis which influenced the main considerations and assumptions selected for the on-farm AD system. As shown in Table 2 the main processes included in all of the analyses are the feedstock acquisition, the construction of the anaerobic digester, purchase of CHP unit, and the sale of electricity and/or heat generated. These processes are the baseline assumptions considered in the reviewed TEAs and LCAs as part of the profitability and mitigation potential of on-farm AD. This boundary in most cases can be limited as it only takes into consideration the main process of AD, namely the production of biogas to generate power without considering the co-products and by-products produced by the process and the environmental benefits of the technology. To characterize the effect of the different variables considered within the boundary of the study for on-farm AD systems, two studies (presented in Table 9) that included both TEA and LCA results with a clear methodology were selected to calculate the abatement cost for small and medium-scale systems. Both studies provided the assumptions considered for the selection of the variables for the TEA and LCA and explained in detail how each scenario was selected for the analysis of the study.

**Table 9.** Case study scenarios for on-farm AD systems in Ireland (small-scale) and the USA (medium scale) for the calculation of the abatement cost and potential of anaerobic digestion.

Scenario	Manure Source	Herd Size	Feedstock (tonnes fresh weight/yr)	Digester Size (m^3^)	CHP (kWe)	Location	Source
S1	Dairy Cows	100	1,070 (Grass Silage) 1,010 (Slurry)	NA	26	Ireland	[30]
S2	Dairy Cows	150	1,199 (Grass Silage) 1,515 (Slurry)	NA	39		
S3	Dairy Cows	200	1,328 (Grass Silage) 2,020 (Slurry)	NA	46		
S4	Dairy Cows	250	1,457 (Grass Silage) 2,525 (Slurry)	NA	55		
S5	Beef Cattle	2,392	3,313 (Corn Husk) 30,558 (Slurry)	3,670	500	US	[31]
S6	Beef Cattle	3,564	3,313 (Rye) 45,515 (Slurry)	3,670	500		
S7	Beef Cattle	3,458	3,313 (Wheat) 44,165 (Slurry)	3,670	500		

Scenarios S1 to S4 are based in Ireland, and scenarios S5 to S7 are based in the USA. While the feedstock differs in both the Irish and US scenarios, both studies represent on-farm AD systems. Typically, in Ireland slurry is collected in open tanks in the winter period for 16 weeks when cattle are housed indoors, the cattle are kept outdoors on pasture during the rest of the year [63]. In the USA, cattle are housed year-round in feedlot operations in which manure is collected periodically based on the amount accumulated and the weather conditions [64]. In the Irish scenarios (S1 – S4), the annual slurry collected is 10.1 t/cow/yr with a total solid content of 8.75% resulting in 0.88 tDM/cow/yr [30]. In the USA scenarios (S5 – S7), the annual manure collected is 12.8 t/cow/yr with a total solid content of 12% resulting in 1.53 tDM/cow/yr [31]. There are obvious differences in the size of the systems assessed; the Irish system can be described as small scale with an electrical output of <100 kW_e_ whilst the US process is of medium-scale (>100 kW_e_) [30].

### 2.8. TEA assumptions

For the calculation of the TEA for scenarios S1 to S7, each of the studies considered different variables for the CapEx, OpEx, and revenues generated by the project to calculate the NPV based on the region of analysis. For both studies, CapEx considered the cost of equipment installation at the AD plant and the cost associated with the CHP electrical plant. The Irish scenarios (S1 – S4) estimated this cost on the average establishment cost based on the installed electrical capacity (kW_e_) of the on-farm AD systems based on reports from Europe [30]. The USA scenarios (S5 – S7) also included in the CapEx the cost for storage of slurry and liquid by-products in the form of a sludge holding tank for the slurry based on the total head of cattle and a lagoon for the liquid effluent produced per head of cattle [31]. The OpEX for both studies accounted for the maintenance, labor, and insurance for the operation of the plant; however, the Irish scenarios (S1 – S4) omitted taxes and interest from the calculation as the authors stated that this would give a distorted representation of the cost of financing [30]. The USA scenarios (S5 – S7) also included the cost of raw materials such as the feedstock acquisition cost (Rye, Wheat, and Corn Stover) and raw manure obtained from cattle feedlot operations, and the waste handling cost for the solid digestate [31].

For revenues, both studies considered incentives as part of the income. The Irish scenarios (S1 – S4) considered two different incentives for energy, the renewable energy FiT scheme for the electricity exported to the grid at a rate of 15.8 c€ kWh^−1^ for CHP with capacity ≤500 kW and the RHI scheme based on the heat produced at a rate of 2.95 c€ kWh^−1^ for AD plants generating less than 300 MWh/yr [30]. For the US scenarios (S5 – S7), the incentives came from the Renewable Electricity Production Tax Credit based on the electricity generated by the system using a cost of $0.015 USD kWhe^−1^. Additionally, this tax credit provides an incentive through a by-product credit claims that provide $35.25 USD per metric ton for solid effluent and $2.64 USD per metric ton for liquid effluent if these are reused as fertilizers on the farm [31]. In addition to the incentives, the Irish scenarios (S1 – S4) consider additional revenues from the sale of exported heat via a district heating system at a rate of 3 c€/kWh taking into consideration the cost of infrastructure and from the cost-saving from avoided electricity and heat use on the farm. The electricity saving is based on the purchase price of 19.9 c€ kWh^−1^ from the electric grid for consumption less than 0.02 GWh/yr and 15.1 c€/kWh for consumption between 0.02 and 0.5 GWh/yr. Meanwhile, thermal energy savings were based on the cost of displacing kerosene as a fuel for heating at a cost of 7.8 c€ kWh^−1^ (based on an energy content of 36.4 MJ/L and a price of 80 c€/L) [30].

### 2.9. LCA assumptions

For the calculations of the net GHG abatement, each of the studies took different approaches based on the processes considered in each LCA methodology. For both the Irish and US scenarios, the overall net GHG abatements (kg CO_2eq_/yr) were calculated by subtracting the total GHG emissions produced by the process minus the GHG savings provided through avoided emissions. The Irish scenarios (S1 – S4) included the GHG emissions produced by the crop production process (grass silage), feedstock collection and transport, biogas production process, and digestate disposal. These emissions were mainly generated from the diesel fuel consumption of the machinery used and from the emissions generated in the combustion of biogas in the CHP unit. The GHG savings were divided between emissions avoided by displacing fossil fuels used for energy generation and avoided fugitive methane emissions from manure storage and manure application to land [30]. The USA scenarios (S5 – S7) considered the GHG emissions produced from the production of feedstock, the feedstock transportation, feedstock preparation for anaerobic digestion, the separation of by-products, and the combustion of biogas in the CHP unit. The GHG savings were based on the avoided emissions from the displacement of chemical fertilizer through the use of digestate as biofertilizer, and from the emissions avoided by displacing fossil fuels used by recycling the heat to the AD process in the form of steam [31].

## 3. Results and discussion

### 3.1. Process variation and the effect on abatement costs for on-farm AD

**Figure 2.**
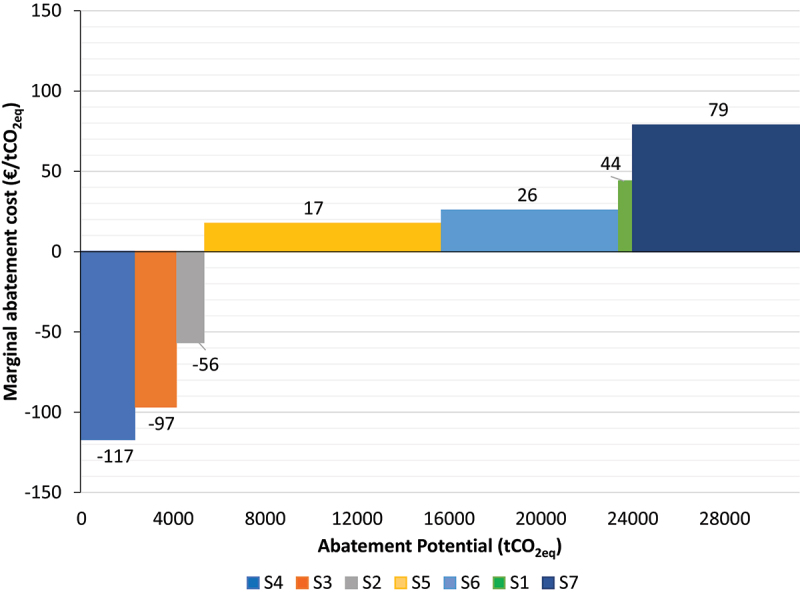
Abatement Cost Curve for small and medium scale on-farm AD systems. Small-scale scenarios S1 to S4 were based on Irish dairy farms with different herd sizes (S1–100, S2–150, S3–200, S4–250) utilizing grass silage and slurry as feedstocks. Medium-scale scenarios S5 to S7 were based on US beef farms with different types of feedstocks (S5- Corn Husk, S6-Rye, S7-Wheat) being co-digested with beef slurry.

Based on the assumptions for both the Irish and US scenarios, the TEA and LCA results reported in each study were used to construct an abatement curve for on-farm AD. The abatement cost of each scenario was obtained as shown in Table 10 by using the IPCC methodology shown in Equation 2 and a discount rate of 4% for the discounted lifetime abatement in guidance with the Irish Public Spending Code [65]. The NPV and net annual GHG savings (t CO_2eq_/yr) were obtained from the TEA and LCA results reported in each study. Box 1 shows in detail the calculations of the abatement cost. A partial approach was selected to present the data. The resulting abatement curve, shown in Figure 2, shows that scenarios S2 to S4 have a negative cost of abatement, making them economically viable as mitigation options, whilst scenarios S1, S5, S6, and S7 have positive abatement costs indicating that they are high-cost abatement solutions.

**Table:** 

Box 1. Calculation for the Abatement Cost Ireland Scenario 1Discounted Lifetime abatement A discount rate of 4% for GHG emissions was assumed for the calculation of the discounted lifetime abatement [65]. A period of 20 years based on the lifetime of the project was assumed. geetha. is the discounted annual abatement in year t and calculated as show in Eq. 3. $DA_{1}=\frac{45}{{\left(1+0.04\right)}^{1}}=\frac{45}{1.04}=43.12\;t\;\;CO_{2}\;eq$. $DA_{2}=\frac{45}{{\left(1+0.04\right)}^{2}}=\frac{45}{1.08}=41.46\;t\;\;CO_{2}\;eq$. . $DA_{20}=\frac{45}{{\left(1+0.04\right)}^{20}}=\frac{45}{2.19}=20.47\;t\;\;CO_{2}\;eq.$ The total project abatement equals the sum of the discounted annual abatement. $DLA=43.12+41.46+\ldots +20.47=609.47\;t\;\;CO_{2}eq$ Abatement cost Using both the NPV reported by the study and the sum of the discounted abatement, the abatement cost curve was calculated using the IPCC methodology in Eq. 2. $AbatementCost=\frac{-\left(-26758\right)}{609.47}=43.9\text{\textbackslash{}euro}\;\;per\;\;t\;\;CO_{2}e$

**Table 10.** Assessment of abatement cost and net GHG abatement for different on-farm AD scenarios.

Scenario	Net GHG abatement (tCO_2eq_ /yr)	Lifetime of the project (Years)	NPV for lifetime of the project (€)	Source	Discounted Lifetime Abatement^d^ (tCO_2eq_)	Abatement Cost (€ per tCO_2eq_)
S1	45	20	−26758^b^	[30]	609	44
S2	88	20	67339^b^		1192	−56
S3	130	20	171168^b^		1773	−97
S4	173	20	275006^b^		2354	−117
S5	599^a^	30	−181076^c^	[31]	10358	17
S6	446^a^	30	−197932^c^		7713	26
S7	421^a^	30	−572124^c^		7273	79

a – Values were obtained by multiplying the GHG emissions reported in the study (kg CO_2_ eq/kWh) by the annual energy generated based on the system capacity of 950kW_e_ with an 85% annual operating capacity (7446 hrs/yr).

b- A discount rate of 5% was used for financing, the tax rate was not considered as part of the calculations of the NPV.

c- An exchange rate of 0.85 USD/€ was used for the NPV values reported in the study to convert values from USD to euro. A discount rate of 7.5% was used for financing while a 39% tax rate was used for income.

d- Based on a discount rate for GHG emissions of 4%.

It is apparent from the results obtained in Figure 2 that the abatement cost of on-farm AD can fluctuate based on the scale of the system, the processes considered, and the system boundary applied for the calculation of the abatement cost. This is evident in scenarios S1 to S7 where the abatement cost ranged between −117 to +79 €/tCO_2eq_, in comparison to the values presented in the Irish *Climate Action Plan* (Table 1) of 115 and 280 €/tCO_2eq_ for biogas to heat and power and biomethane, respectively [20]. The assumptions selected for each study and the system boundary considered determine the overall result of the financial viability and the environmental mitigation of the system. Scenarios evaluated in the *Climate Action Plan* considered large-scale AD scenarios in which the GHG savings came mainly from displacing fossil fuels and the income was derived from the sale of the energy generated. The income for the biogas to heat and power scenario was generated from the sale of the energy set between 6 and 8 c€/kWh and from the renewable energy FIT of 14 c€/kWh. The biomethane scenario only considered the income derived from the sale of the upgraded gas as biomethane at 3.5 c€/kWh. It is unclear to the authors how the cost per tonne of CO_2_ abated was calculated for each scenario as there is not enough insight on the methodology that was used to calculate the cost of each measure in the *Climate Action Plan* analysis.

For the scenarios considered in this paper for Ireland (S1 – S4) and the USA (S5 – S7), the system boundary selected for both TEA and LCA considered additional sources of income and GHG savings which were likely not considered by the analysis completed for the Climate Action Plan and may result in a lower cost of abatement. These include GHG savings from avoided fugitive methane emissions from manure storage and manure application to land and avoided emissions from the displacement of chemical fertilizer from the use of digestate as biofertilizer. In the case of revenues, these include the sale of heat in district heating networks; incentives from the management of by-products from digestate; and the offset of current heat and/or electricity costs at the farm. When incentives are included for the calculation of the abatement cost, it allows investors, the system operators, and policy makers, to see the magnitude of mitigation that each technology can achieve, as well as the economic performance of the system from the perspective of the system operator.

In the case of the scenarios for Ireland (S1-S4), more sources of revenues were considered (incentives, sale of heat produced, cost-savings from displaced energy) which resulted in a lower cost of abatement as the additional sources of income resulted in a positive NPV in comparison to the scenarios for the USA (S5-S7) that considered fewer sources of revenue (tax credit incentives) and additional costs (feedstock acquisition cost and taxes) which resulted in a negative NPV. The LCA of both studies considered similar sources of GHG emissions. In the US scenarios, the GHG emissions savings included for displacing synthetic fertilizer with digestate (as a biofertilizer) which resulted in greater mitigation owing to the extended system boundary in comparison to the Irish scenarios. The Irish scenarios only considered the GHG savings from the avoided fugitive methane emissions from manure storage and manure application to land. The system boundary differs between each study assessed and can vary from case to case based on the methodology used for the calculation of the TEA and LCA. This will alter the abatement cost which makes selecting a single point value to represent a mitigation technology difficult.

MACCs are a standard policy tool to assess the cost-effectiveness of mitigation options in a simplified and straightforward manner; however, the MACC results for certain technologies may present a single average cost that does not account for system variation, which ultimately may affect the abatement cost and the abatement potential of a mitigation technology. In particular, MACC analyses that use a partial approach have shortcomings that include a lack of disclosure of the input data assumptions selected for the calculations, the lack of consideration of interdependencies and interactions between variables, and the limited representation of uncertainty [66]. These shortcomings can be a result of the amount of data required for the construction of the curve itself and the methodology selected for the calculation.

### 3.2. Integration of uncertainty analysis for the calculation of the abatement cost

Recent MACC values in other sectors have started to consider the representation of uncertainty during the construction of MACCs to provide a more transparent methodology where the cost and abatement potentials are compared [67–69]. To carry out this methodology allowing for uncertainty, the single data points used in the construction of the curve need to be sampled from probability distributions, which in most cases can be performed using Monte Carlo simulations. However, incorporating various uncertainties may complicate the overall modeling of the abatement cost and potential, so it is recommended only to include the uncertainties of the input parameters that directly affect these values; these parameters can be obtained with a sensitivity analysis. In particular, the discount rate selected is a parameter that needs to be assessed in the sensitivity and uncertainty analysis as it affects both the calculations of the abatement cost and potential. Furthermore, given the shortcomings of the partial approach, the inclusion of uncertainty analysis will improve the robustness of the results illustrated in MACCs by providing the potential range of abatement and abatement costs the mitigation technology provides.

### 3.3. Inclusion of economic diversification and the environmental benefits of on-farm AD

Only a handful of TEAs reviewed recognized two or more of the additional financial benefits of on-farm AD which may include the income from the sale of biofertilizers and co-products, the reduction of energy costs (for farm equipment, lighting, heating) and the reduction in chemical fertilizer use [24,31,32]. Including more inputs and outputs in the system boundary provides a more representative methodology that can include possible additional financial benefits of the technology and provide a more realistic estimate of the viability of on-farm AD.

A recent TEA by González-Arias et al. [70] found that biogas plants with a digester size of 100 and 250 m^3^ were unprofitable in comparison to larger biomethane production plants where the only revenue considered was the sale of biomethane (which depends on subsidies and incentives available). However, the same study did acknowledge that the profitability of small plants can be improved by considering other revenue streams where waste is converted into value-added materials and energy. Furthermore, new approaches for on-farm AD systems may provide extra revenues that can improve profitability by expanding the system boundary to include additional processes. Examples of this can be seen in biorefineries for isobutene and xylo-oligosaccharides evaluated by Lopes et al. [71] or the use of CO_2_ produced as a by-product of AD for tomato cultivation in greenhouses evaluated by Oreggioni et al. [72] which were both proven to be economically viable. Another source of income could be waste management fees by integrating bio-stabilized organic solid wastes diverted from landfills as feedstock to the anaerobic digestion process which could increase the financial viability of the system [73]. The integration of such fees has also been explored in Ireland as part of the National Waste Management Plan, in which waste fees could be modulated for users in a ‘pay as you throw’ scheme based on the amount of waste delivered to the waste management system [74]. However, the acceptance of bio-stabilized waste as an additional feedstock by farm scale anaerobic digestion systems would need to be strict in accordance with all health and safety requirements, specifically those governing the operation of anaerobic digestion plants to avoid the unintended spread of pathogens (with reference to CN9 and CN11 from the Department of Agriculture, Food and the Marine) [75].

For LCAs, the main emissions savings considered for the calculation of the GHG emissions comes from the emissions avoided from manure management, displacement of farm energy demand and energy exported, and displacement of fertilizer as shown in Table 8. Although there are certain environmental improvements that are not linked to GHG emissions such as the odor reduction through the use of biofertilizer and the improvement of water quality by reducing eutrophication, these were not considered in any of the analyses [76]. This environmental benefit of AD could also be included within the system boundary if the GHG emissions that would be produced by technologies used to treat such problems are included in the calculations within the system boundary. This could prove beneficial in order to compare scenarios where AD is not applied as seen in a MACC produced for the UK Climate Committee [62]. This particular MACC considered the environmental mitigation of AD as part of the assumptions based on the treatment of livestock excreta that would otherwise be stored in slurry tanks or lagoons. Furthermore, integrating technologies such as pyrolysis could allow anaerobic digestion systems to offer atmospheric carbon removal services based on the production of biochar from digestate [77]. This could provide an alternative use for excess digestate produced in the anaerobic digestion process, which might not be utilized entirely by the farm. If all possible financial benefits and environmental improvements are considered within the system boundary, a more representative abatement cost and mitigation potential of anaerobic digestion, and indeed any technology, can be obtained.

## 4. Limitations of work

As part of this work, Ireland was presented as an example region to compare the abatement cost previously calculated for on-farm anaerobic digestion in the Climate Action Plan 2019 with two case studies which presented results of on-farm anaerobic digestion scenarios at different scales. The calculation of the abatement cost can vary based on the region of analysis, or the methodology selected in undertaking the MACC assessment. The data used in this work were explicitly obtained to compare the methodology and system boundary applied in different regions for the calculation of key parameters such as the NPV and emissions savings, which allow the identification of all the potential financial benefits and environmental improvements that anaerobic digestion can provide. The methodology in this work can be expanded to other regions to expand the current system boundary used for the calculation of the abatement cost of a technology, in this case, anaerobic digestion. This can aid the representation of the abatement cost of anaerobic digestion whilst accounting for externalities such as improved manure management practices which are not commonly represented in abatement cost curve analyses.

## 5. Conclusions and recommendations

### 5.1. Conclusions

To assess the financial viability of on-farm anaerobic digestion, the key parameters to consider in a techno economic assessment (TEA) are the net present value (NPV), the internal rate of return (IRR), and the payback period. Likewise, to assess the environmental impact, the key consideration in a lifecycle assessment (LCA) for the GHG assessment is to include emissions savings from the displacement of fossil fuel from avoided energy use; the displacement of chemical fertilizer use on-farm; and the emissions avoided from manure management. Most of the literature reviewed provided results from a TEA and LCA but did not calculate the abatement cost based on the results of the TEA and LCA.

Within this study, the abatement cost was evaluated for seven different scenarios for on-farm anaerobic digestion at different scales (small and medium) based on literature case studies (which included clear definitions of the methodology used for the TEA and LCA). Each scenario considered different processes and boundaries for both TEA and LCA which provided a range of different values of abatement cost and potential. The abatement cost varied from −117 €/tCO_2eq_ to +79 €/tCO_2eq_ clearly indicating that the variables selected for the study have a significant influence on the results. Additionally, the scale of the on-farm AD system and the revenue streams considered influenced the results. Small on-farm AD systems that accounted for a larger number of revenue streams (Irish scenarios) had a better economic performance but lower abatement potential than larger systems (US scenarios) in the studies analyzed which resulted in lower abatement costs. On the contrary, scenarios with larger on-farm AD systems achieved greater GHG mitigation owing to the increased system size despite the low economic performance of the systems. Including GHG emissions savings from the avoided use of fertilizer further reduced abatement costs in the US scenarios. As such, accounting for additional income and emission savings from co-benefits can reduce the abatement cost.

Considering different variables within a study has a substantial impact on the calculation of the cost and abatement potential of on-farm anaerobic digestion. The scale and processes considered within the analysis determine the overall result in terms of financial viability and environmental mitigation. The system boundary directly influences the processes considered and therefore influences the results of the calculation. In the scenarios assessed in this work, it was found that if the study considered additional sources of revenue streams and savings (therefore a larger number of processes) a lower abatement cost would be obtained. Furthermore, the methodology differs between each study, and the processes considered to calculate the cost and abatement can vary from case to case based on the methodology and assumptions of each study. Prior analyses which presented a single value for the abatement cost for on-farm AD may not be able to accurately capture this variability in the system boundary considered; the scale of the system; and the revenue streams considered.

### 5.2. Recommendations

The authors recommend that the development of MACCs that include AD as a mitigation technology should be modified to account for input parameter uncertainty and the resulting uncertainty in the abatement cost and mitigation potential. This could aid policy-makers when developing energy policies in providing a better representation of the range of abatement cost values for on-farm AD, as well as in highlighting the uncertainty within different system scales and processes considered within the system boundary. For future research, it is recommended by the authors that the assessment of the abatement cost of on-farm AD should use a combined analysis tool in which the TEA and LCA are carried out simultaneously. It is further recommended that this combined tool should consider all the relevant inputs and outputs for the abatement cost calculations and should incorporate sensitivity analysis and uncertainty analysis to provide a more realistic assessment of on-farm AD systems.

## Abbreviations

AD: Anaerobic Digestion
CapEx: Capital Expenditure
CHP: Combined Heat and Power
CH_4_: Methane
CO_2_: Carbon Dioxide
EU: European Union
FIT: Feed-in Tariffs
GHG: Greenhouse Gas
GBP: British Pound Sterling
kW_e_: Kilowatt-electric
kWth: Kilowatt-thermal
kWh: Kilowatt-hour
ILCD: International Reference Life Cycle Data System
IRR: Internal Rate of Return
LCA: Life-Cycle Assessment
MACC: Marginal Abatement Cost Curve
MWh: Megawatt-hour
MW_e_: Megawatt-electric
NPV: Net present Value
REDII: Recast of the Renewable Energy Directive
RHI: Renewable Heat Incentive
TEA: Techno-economic Assessment
OpEX: Operational Expenditure

## Data Availability

The authors confirm that the data supporting the findings of this study are available within the article [and/or] its supplementary materials.
